# Fetomaternal Hemorrhage following Placement of an Intrauterine Pressure Catheter: Report of a New Association

**DOI:** 10.1155/2015/348279

**Published:** 2015-08-31

**Authors:** Fadi G. Mirza, Harshwardhan M. Thaker, Wendy L. Flejter, Mary E. D'Alton

**Affiliations:** ^1^Division of Maternal-Fetal Medicine, Department of Obstetrics and Gynecology, Columbia University Medical Center, New York, NY 10032, USA; ^2^Division of Maternal-Fetal Medicine, Department of Obstetrics and Gynecology, American University of Beirut Medical Center, Beirut, Lebanon; ^3^Department of Pathology, Primary Children's Medical Center, Salt Lake City, UT 84113, USA; ^4^Department of Genetics, Caris Diagnostics, Phoenix, AZ 85040, USA

## Abstract

Fetomaternal hemorrhage (FMH) can be associated with significant perinatal mortality. Our review of the literature did not identify any cases of FMH following placement of an intrauterine pressure catheter (IUPC). In our case, an IUPC was inserted in a patient undergoing induction of labor at term. Fetal bradycardia ensued shortly after placement, warranting an emergent cesarean delivery. Severe neonatal anemia was identified, and evaluation of maternal blood was consistent with massive FMH. This is the first reported association between FMH and IUPC placement. If this relationship is validated in future reports, appropriate changes in clinical practice may be warranted.

Fetomaternal hemorrhage (FMH) occurs when fetal blood passes into the maternal circulation at any stage of pregnancy. FMH can be associated with significant perinatal mortality, primarily related to severe anemia and hypotension in the fetus or newborn. A number of predisposing factors have been identified. Our review of the literature did not identify any cases of FMH following insertion of an intrauterine pressure catheter (IUPC), a potential new association that we report in this paper.

A 24-year-old nulliparous female was followed up at our perinatal clinic secondary to known factor VII deficiency, a diagnosis that was made during pregnancy following the incidental finding of an abnormal prothrombin time (PT). The patient presented for induction of labor at 41 weeks of gestation for postdates and new-onset preeclampsia. The patient reported good fetal movement. On admission, the cervix was noted to be 1 cm dilated and 50% effaced, and the fetal heart tracing was consistent with category 1. Laboratory workup showed a normal complete blood count. The coagulation profile showed a partial thromboplastin time (PTT) of 28.3 seconds, a prothrombin time (PT) of 22.8 seconds, and an international normalized ratio (INR) of 1.90. Cervical ripening was initiated with misoprostol, a synthetic prostaglandin E1 analog, followed by mechanical dilation of the cervix using an intracervical Foley balloon catheter. The patient was continuously monitored using cardiotocography (CTG) and tocodynamometer before and after misoprostol placement. The cervix was noted to be 3 cm dilated and oxytocin intravenous drip was subsequently started and amniotomy was performed. The patient was maintained on magnesium sulfate for seizure prophylaxis. Furthermore, recombinant factor VII was administered intravenously at a dose of 2400 mcg every four hours in consultation with the hematology and blood bank services. Pain management was achieved using remifentanil PCA at the discretion of the anesthesiology team secondary to the finding of scoliosis on physical examination. The oxytocin infusion rate was gradually increased and, approximately 5 hours later, an intrauterine pressure catheter (IUPC) was inserted without difficulty at 3:28 AM in order to achieve more adequate monitoring. It is noteworthy that there was no evidence of tachysystole. At 3:30 AM, fetal heart tracing could not be documented using the previously utilized external monitor. At 3:43 AM, a fetal scalp electrode was successfully placed which demonstrated fetal bradycardia with a fetal heart rate in the range of 30 s–40 s. The patient was promptly transferred to the operating room for an emergent cesarean delivery that was performed under general endotracheal anesthesia. The timeline is presented in Table 1.

A live born male was delivered at 3:54 AM, weighing 3750 grams with an Apgar score of 0 and 0 at 1 and 5 minutes, respectively. Arterial cord blood gases were essentially unremarkable with a pH 7.24, pCO_2_ 57, HCO_3_ 25, and base deficit 3. With the pediatric team in attendance, the infant was delivered floppy and pale, without spontaneous respiration or discernible heart rate by auscultation. Cardiopulmonary resuscitation was initiated. The initial complete blood count noted severe anemia with hematocrit of 23%. In view of the maternal history, factor VII levels were assayed and reported as suboptimal at 17% (normal range 50–150%). The decisions were subsequently made to withdraw supportive measures, and the infant was pronounced dead at approximately 8 hours of life. An autopsy was pursued which showed no gross abnormalities. Pathologic examination of the umbilical cord and placenta revealed no gross vascular anomalies, aneurysms, or thrombi. Microscopic examination revealed a small number of macrophages within the membranes that contained pigment consistent with meconium. There was no significant increase in the number of fetal nucleated red cells (NRBC) in the fetal vessels or in the intervillous space. Finally, there were no placental infarcts, intervillous thrombi, or retroplacental hematomas.

Postoperatively, a maternal blood sample was promptly sent for a Kleihauer-Betke (KB) assay. The latter returned elevated at a level of 4.6%, which corresponds to a loss of about 166–230 cc of fetal whole blood. This variation is a reflection of different assumptions regarding the average maternal blood volume, the average maternal hematocrit, and the average fetal hematocrit [1]. Additional testing of a maternal blood sample identified 101.5 NRBC per milliliter of maternal blood. Of interest, Huang and colleagues showed that the number of NRBC/mL maternal blood from an unselected population of presumed normal singleton pregnancies has a mean of 37.68 and a median of 21.08 [2]. Hence, the NRBC count in our case indicates a threefold increase in circulating NRBC over normal pregnancies and, like the KB result, is consistent with FMH.

The reported incidence of FMH appears to vary from one source to another, although a study by Bowman and colleagues identified FMH in up to 75% of pregnancies [3]. There is no universal definition for massive FMH and cutoff volumes that range from 10 to 150 mL have been reported in the literature [4]. The incidence thus varies from 1 to 3 per 1,000 births, depending on which cutoff is utilized [5]. The underlying etiology remains unknown in the majority of cases. Like the majority of cases of FMH, the underlying etiology remains unknown. In this paper, a clear cause was not identified in over 80% of cases of massive FMH. Predisposing factors include placental and cord abnormalities, maternal trauma, and obstetric procedures or operations. Unfortunately, known risk factors do not reliably predict massive FMH, and it is likely that future research will identify a number of previously identified risk factors [6].

The standard quantitative evaluation for FMH has traditionally been the KB screen [7]. This test relies on the fact that hemoglobin F, the major component of fetal erythrocytes, is more resistant to acid elution than the adult-life hemoglobins. Hence, the hemoglobin F-containing erythrocytes appear pink under the microscope while adult erythrocytes appear colorless. This discrepancy in appearance allows the operator to count fetal cells and report them as percentage of the adult cells. One of the main limitations of this test is the relatively long turnaround time, which can be in the order of days. Furthermore, the KB screen remains a subjective test as the quality of the staining process and the correct identification of fetal cells are highly dependent upon the technician's skill. Thus, several studies published since the introduction of the KB assay have demonstrated poor reproducibility [8, 9]. Another major limitation to the use of a KB test is the imprecision in translating the reported percentage of fetal cells into a volume of blood lost. The latter can vary with different assumptions regarding the average maternal blood volume, maternal hematocrit, and fetal hematocrit. For instance, a KB screen of 3% corresponds to an estimated fetal blood volume that ranges from 108 cc to 150 cc [1].

More recently, other techniques have emerged to isolate and enumerate NRBC as a potential resource for monitoring and diagnosis of maternal, fetal, and neonatal diseases. In our case, NRBC were measured using a microfluidics-based approach. This technique can be performed on 5 to 10 mL of maternal blood, is highly reproducible, and can be carried out in approximately 6 hours. It involves a two-step enrichment process designed to separate blood cells into a nucleated fraction and a nonnucleated fraction and then into a hemoglobin-containing fraction and a non-hemoglobin-containing fraction [2]. Cells collected by this process are spun onto slides that are then analyzed to identify nucleated cells. Target NRBC are subsequently confirmed manually on a BioView Duet microscope.

To our knowledge, FMH has not been reported secondary to IUPC placement. While a definitive relationship could not be established in our case, we recognized a temporal relationship between IUPC insertion and an immediate change in fetal heart rate with a subsequent diagnosis of FMH. Such a profound change in fetal testing following foreign body placement is not unique to this case. A recent NICHD-sponsored study utilized a fetal oxygen sensor that was inserted into the uterine cavity after placement of an internal fetal heart rate monitor and IUPC [10]. Out of 5553 subjects who consented to this study, 54 patients sustained prolonged fetal heart rate deceleration, 14 of which underwent an immediate cesarean delivery. There was no evidence of uterine perforation or placental abruption in any of the cases. The investigators proposed direct impingement of the sensor on the umbilical cord or manipulation of the fetal head as a possible explanation for this complication. While this may represent a plausible explanation, the uniqueness of our case lies in the identification of FMH following IUPC placement.

The underlying pathophysiology in our case is not fully understood. Fetal susceptibility due to an inherent factor VII deficiency remains a potential risk factor for the observed massive FMH. Moreover, in view of the known association between FMH and mechanical factors like abdominal trauma and external cephalic version, a plausible explanation can be an intrauterine pressure change that was triggered by the placement of the IUPC. While pathologic examination of the placenta and cord failed to reveal gross vascular aneurysms or anomalies, it remains possible that there were smaller microscopic lesions of fetal vessels that may have escaped sampling when microscopic sections were taken. Such lesions would be particularly difficult to identify since they are expected to collapse following an episode of FMH.

In conclusion, FMH can be associated with significant perinatal mortality. Although numerous risk factors have been identified, the majority of cases occur in the absence of identifiable causes. It is likely that ongoing and future research will recognize additional factors contributing to this phenomenon. The occurrence of FMH following an intrauterine foreign body insertion such as an IUPC may be underestimated. In this paper, we reported the first association between massive FMH and IUPC placement. If this relationship is validated in future reports, appropriate changes in clinical practice may be warranted. Such modifications may include strong consideration of a fetal scalp electrode prior to catheter insertion, ultrasound guidance during its insertion, and/or clear documentation of the fetal heart rate and the presence of the provider in close proximity to the patient in the immediate postinsertion period.

## Figures and Tables

**Table 1:** Sequence of events.

Time	Activity
9:30 AM	Placement of misoprostol 25 mcg vaginally in tablet form
1:30 PM	Insertion of Foley balloon catheter
5:00 PM	Initiation of oxytocin drip
10:45 PM	Amniotomy
3:28 AM	Insertion of IUPC
3:43 AM	Insertion of FSE
3:54 AM	Delivery via cesarean section
